# Utilizing the Brain Vasculature to Improve Delivery and Safety of the Next Generation of AD Therapeutics

**DOI:** 10.1002/alz70859_102246

**Published:** 2025-12-25

**Authors:** Joy Zuchero

**Affiliations:** ^1^ Denali Therapeutics, South San Francisco, CA USA

## Abstract

**Background:**

Although the first generation of immunotherapies for Alzheimer’s disease (AD) are now clinically approved, amyloid‐related imaging abnormalities (ARIA) remain a major safety problem for this class of drugs.

**Method:**

Here, we report an antibody transport vehicle (ATV) targeting the transferrin receptor (TfR) for brain delivery of amyloid beta (Aβ) antibodies that significantly reduced ARIA‐like lesions and improved plaque target engagement in a mouse model of amyloid deposition.

**Result:**

Asymmetrical Fc mutations (ATVcisLALA) allowed the molecule to selectively retain effector function only when bound to Aβ while mitigating TfR‐related hematology liabilities. Mice treated with ATVcisLALA:Aβ exhibited broad brain parenchymal antibody distribution; in contrast, anti‐Aβ IgG was highly localized to arterial perivascular spaces where vascular Aβ accumulates and likely plays a role in induction of ARIA. Importantly, ATVcisLALA: Aβ almost completely eliminated ARIA‐like lesions and vascular inflammation associated with anti‐Aβ treatment.

**Conclusion:**

Taken together, ATVcisLALA has the potential to significantly improve both safety and efficacy of Aβ immunotherapy through enhanced biodistribution mediated by transport across the blood‐brain barrier.

